# Immune-mediated platelet depletion augments Alzheimer’s disease neuropathological hallmarks in APP-PS1 mice

**DOI:** 10.18632/aging.204502

**Published:** 2023-02-01

**Authors:** Diana M. Bessa de Sousa, Ariane Benedetti, Barbara Altendorfer, Heike Mrowetz, Michael S. Unger, Katharina Schallmoser, Ludwig Aigner, Kathrin Maria Kniewallner

**Affiliations:** 1 Institute of Molecular Regenerative Medicine, Paracelsus Medical University, Salzburg, Austria; 2 Spinal Cord Injury and Tissue Regeneration Center Salzburg (SCI-TReCS), Paracelsus Medical University, Salzburg, Austria; 3 Institute of Experimental Neuroregeneration, Paracelsus Medical University, Salzburg, Austria; 4 Department of Transfusion Medicine, University Clinic, Paracelsus Medical University, Salzburg, Austria; 5 Austrian Cluster for Tissue Regeneration, Vienna, Austria

**Keywords:** Alzheimer’s disease, platelets, amyloid-beta, microglia, astrocytes

## Abstract

In Alzheimer’s disease (AD), platelets become dysfunctional and might contribute to amyloid beta deposition. Here, we depleted platelets in one-year-old APP Swedish PS1 dE9 (APP-PS1) transgenic mice for five days, using intraperitoneal injections of an anti-CD42b antibody, and assessed changes in cerebral amyloidosis, plaque-associated neuritic dystrophy and gliosis. In APP-PS1 female mice, platelet depletion shifted amyloid plaque size distribution towards bigger plaques and increased neuritic dystrophy in the hippocampus. In platelet-depleted females, plaque-associated Iba1+ microglia had lower amounts of fibrillar amyloid beta cargo and GFAP+ astrocytic processes showed a higher overlap with thioflavin S+ amyloid plaques. In contrast to the popular hypothesis that platelets foster plaque pathology, our data suggest that platelets might limit plaque growth and attenuate plaque-related neuritic dystrophy at advanced stages of amyloid plaque pathology in APP-PS1 female mice. Whether the changes in amyloid plaque pathology are due to a direct effect on amyloid beta deposition or are a consequence of altered glial function needs to be further elucidated.

## INTRODUCTION

Alzheimer’s disease (AD) is a neurodegenerative disease associated with progressive and irreversible memory loss and cognitive decline. It represents the most common form of dementia and has a complex and multifactorial pathology [1]. Classically, AD is characterized by the presence of two types of misfolded protein aggregates in the brain: amyloid beta plaques and neurofibrillary tau tangles. However, several other neuropathological features are found in AD brains, including neuronal and synaptic loss, neuroinflammation, and cerebrovascular dysfunction [2, 3]. The exact etiology of this disease is still poorly understood and there is yet no curative treatment for it [1], resulting in a pressing need to better understand the underlying disease mechanisms of AD.

Amyloid beta deposition is a central event in AD pathogenesis linked to neuronal damage and neuroinflammation [4]. Amyloid beta peptides are formed by proteolytic cleavage of the amyloid precursor protein (APP) (reviewed elsewhere [5, 6]) and aggregate not only in the brain as amyloid plaques but also in the wall of cerebral blood vessels, originating a condition known as cerebral amyloid angiopathy (CAA) [7, 8]. In the brain, amyloid plaques are typically surrounded by a halo of dystrophic neurites – abnormally swollen axonal and dendritic processes massively filled with lysosomal-like structures [9] that are associated with neuronal loss [8]. Furthermore, amyloid beta aggregates are enclosed by reactive microglia and astrocytes [10], which limit plaque growth and toxicity [11, 12]. However, sustained activation of glial cells over time appears to fuel neuroinflammation and enhance neuronal cell death, contributing to AD progression [13, 14].

For a long time, amyloid beta in the brain was thought to be originated in the brain itself [15]. However, several peripheral tissues and cells also produce amyloid beta peptides [16–20], which might enter the brain and cause cerebral amyloidosis, neuroinflammation and cognitive deficits [15, 21]. Platelets are the primary peripheral source of amyloid beta [21, 22], contributing to about 90% of the amyloid beta peptides circulating in the human blood [22]. They express APP and produce amyloid beta peptides [16, 17], which are secreted upon platelet activation [17]. In AD, platelets have an increased activation status [23] and might contribute to cerebrovascular and cerebral amyloid beta deposition [24–29].

In transgenic AD mice, platelets contribute to the onset and development of CAA [24, 26, 27, 29]. Platelets aggregate at vascular amyloid deposits [28] and promote amyloid beta deposition by releasing clusterin to promote amyloid beta fibrillization and adenosine diphosphate (ADP) to enhance platelet activation [24]. Interestingly, in APP-Swedish Dutch Iowa mice, platelet aggregation in brain vessels precedes the appearance of amyloid pathology in the brain [27] and platelets derived from this mouse model damage cortical brain vessels and induce vascular amyloid deposition and neuroinflammation when transfused into wild-type mice [26]. Similarly, the transfusion of transgenic APP-PS1 mouse platelets into wild-type mice causes cerebral amyloidosis, neuroinflammation and cognitive deficits in the recipient mice [25].

The potential role of platelets in amyloid beta deposition led to the hypothesis that reducing platelet numbers might ameliorate AD pathology [30]. Here, we performed immune-mediated platelet depletion in APP-PS1 mice with an already fully developed amyloidosis and investigated its effects on classical hallmarks of AD: amyloid plaque pathology, plaque-associated neuritic dystrophy and gliosis.

## RESULTS

### Anti-CD42b antibody injection induces platelet depletion in APP-PS1 mice

**Figure 1:**
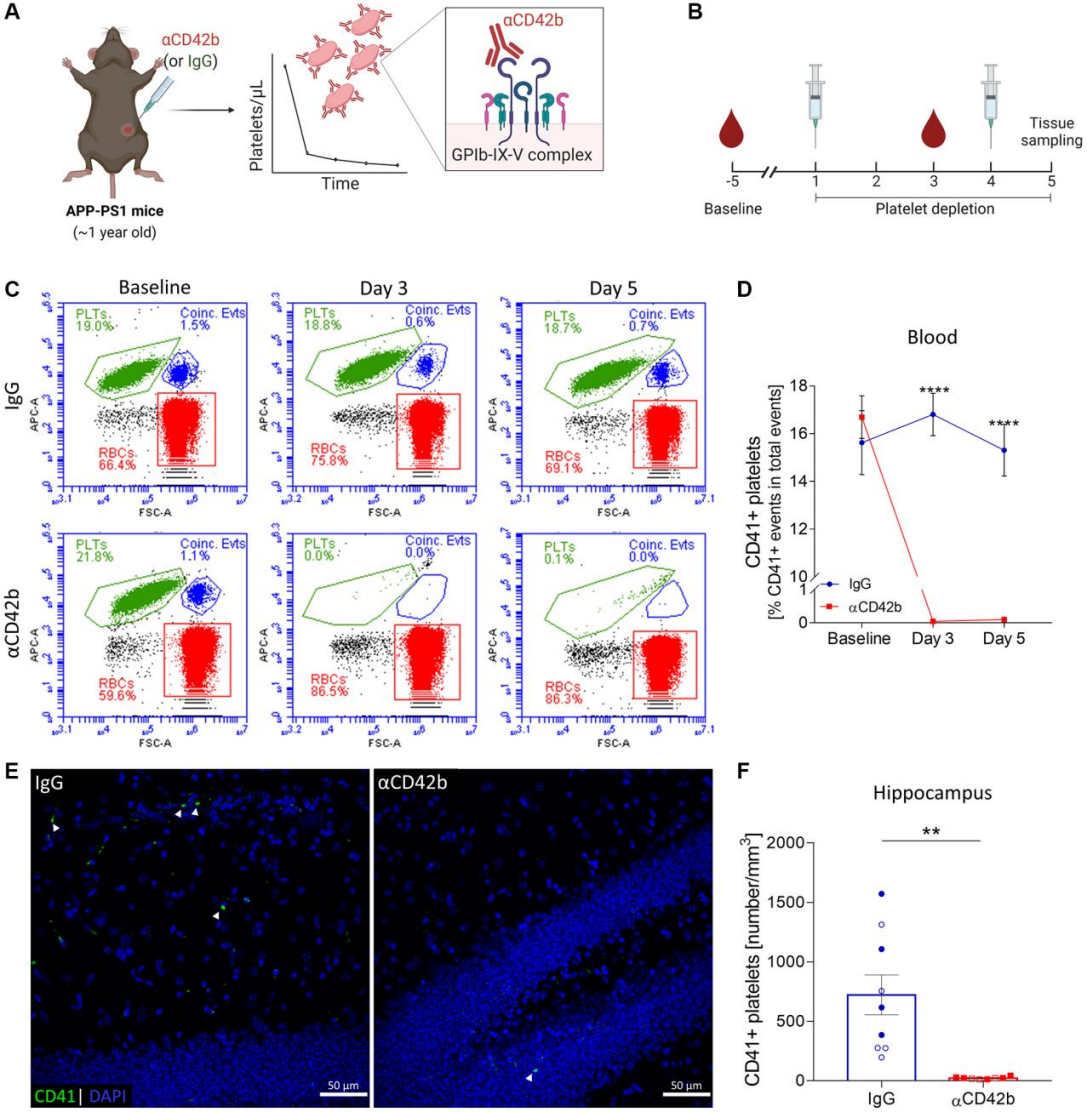
**Immune-mediated platelet depletion in APP-PS1 mice.** (**A**) APP-PS1 mice were intraperitoneally injected with an anti-CD42b antibody (αCD42b) to induce thrombocytopenia. The injection of non-immune rat immunoglobulins (IgG) was used as a control. (**B**) Experimental setup. (**C**) Platelet depletion efficacy was monitored by whole-blood flow cytometry using an allophycocyanin (APC)-labelled anti-mouse CD41 antibody to label platelets (PLTs). Representative flow cytometry plots and gate strategy. (**D**) Anti-CD42b antibody administration effectively reduced blood platelet counts. (**E**) Brain tissue immunolabelled for CD41 (green) was used to quantify platelets in the hippocampus. DAPI (blue) was used as nucleus staining. (**F**) Platelets were significantly reduced in the hippocampus of platelet-depleted mice. Data are shown as mean ± SEM. Statistical analysis was performed by two-way ANOVA with Holm-Šídák’s multiple comparisons test (**D**: *n* = 8–9/treatment) and unpaired Student’s *t* test (**F**: *n* = 8–9/treatment; “full forms” represent females and “empty forms” males). ^****^p < 0.0001; ^**^p < 0.01. Scale bar: 50 μm. (**A**, **B**) Created with Biorender. Abbreviations: PLTs: platelets; RBCs: red blood cells; Coinc. Evts: Coincident platelet and red blood cell events.

To investigate the contribution of platelets to AD pathogenesis, we induced thrombocytopenia in one-year-old APP-PS1 mice. At this age, APP-PS1 mice show severe amyloid plaque pathology, gliosis, cerebrovascular alterations, and cognitive deficits [31–37]. We intraperitoneally injected mice with a commercially available antibody preparation directed against CD42b – a glycoprotein (GP) part of the GPIb-IX-V complex, a pivotal platelet surface receptor for hemostasis [38]. Antibody-mediated targeting of CD42b promotes a quick and efficient Fc-independent platelet clearance in mice, as previously demonstrated by others [39–42] (Figure 1A, 1B). To assess the efficiency of platelet depletion in our mouse model, we monitored platelet counts by flow cytometry using APC-CD41 immunolabeled blood samples (Figure 1C). Injection of anti-CD42b antibody (αCD42b) reduced blood circulating platelets up to 99% compared with baseline, whereas injection of non-immune rat immunoglobulins (IgG) did not affect platelet counts (Figure 1C, 1D). Mice treated with αCD42b also showed significantly reduced numbers of platelets in brain tissue (i.e., hippocampus and cortex) compared with control IgG treated mice (Figure 1E, 1F and Supplementary Figure 1).

As severe thrombocytopenia is associated with a hemorrhagic phenotype, to assess the occurrence of spontaneous bleedings, we analyzed complete blood counts on days 3 and 5 (Supplementary Tables 1, 2) and monitored mice for signs of lethargy and pain. On day 3 (48 hours after the first antibody injection), platelet-depleted mice showed increased bleeding after venipuncture for blood collection (which was stopped by applying firm and prolonged pressure to the puncture site). Besides severe thrombocytopenia, platelet-depleted mice presented a lower mean corpuscular hemoglobin concentration (MCHC) than control IgG treated mice (Supplementary Table 1) without additional hematological alterations. On day 5, platelet-depleted mice showed lower red blood cell counts (RBC) and hemoglobin concentration (Supplementary Table 2) and higher mean corpuscular volume (MCV) than control mice. However, we observed no signs of subcutaneous or internal bleeding by visual inspection of the skin or inner organs during tissue sampling. Platelet-depleted mice appeared more lethargic than control mice but showed no evident signs of ongoing pain based on the Grimace pain scale.

Female mice are reported to tolerate thrombocytopenia better than males [39]. To assess whether female and male APP-PS1 mice reacted differently to αCD42b injections, we investigated sex-specific effects on hematological parameters, body weight and platelet depletion efficiency in brain tissue. Females and males showed similar hematological changes in response to the treatment (Supplementary Tables 1, 2). Females in both experimental groups showed weight loss (Supplementary Table 2), whereas only platelet-depleted males lost weight during the experiment. The efficiency of platelet depletion in the brain was similar between females and males, with platelet-depleted mice showing about 97% lower platelet numbers in the brain compared with sex-matched IgG treated mice (hippocampus: females: IgG = 920.3 ± 528.9 platelets/mm^3^ versus αCD42b = 26.6 ± 10.88 platelets/mm^3^; males: IgG = 563.4 ± 473.8 platelets/mm^3^ versus αCD42b = 16.67 ± 5.51 platelets/mm^3^).

### Platelet depletion shifts amyloid plaque size distribution in APP-PS1 females

**Figure 2:**
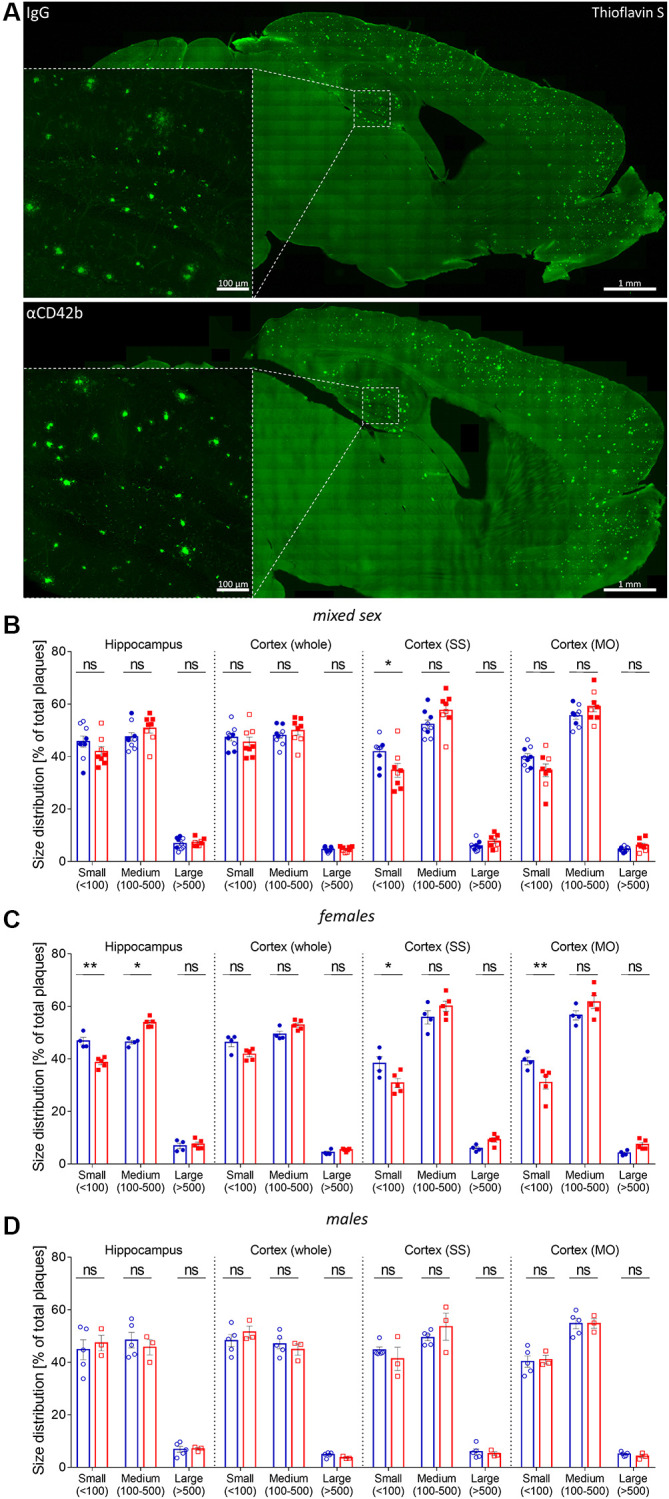
**Platelet depletion shifts plaque size distribution in APP-PS1 females.** (**A**) Amyloid plaque pathology was assessed in thioflavin S stained brain sections. (**B**) Plaque size distribution (% of small, medium, and large plaques) was analyzed in the hippocampus, whole cortex, somatosensory (SS) and somatomotor (MO) cortex. (**C**) APP-PS1 platelet-depleted females showed a shift in the percentage of small (30–100 μm^2^) and medium-size (100–500 μm^2^) amyloid plaques in the hippocampus and a reduced percentage of small-size amyloid plaques in SS and MO. (**D**) No differences were observed in platelet-depleted males. Data are shown as mean ± SEM. Statistical analysis was performed by ordinary two-way ANOVA with Tukey’s multiple comparisons test (*n* = 8–9/treatment; “full forms” represent females and “empty forms” males). ^**^p < 0.01; ^*^p < 0.05. Scale bar: 1 mm (insert 100 μm).

Platelets are one of the major sources of blood circulating amyloid beta [21, 22], and platelet-derived amyloid beta seems to be involved in the formation of cerebrovascular [24, 26] and cerebral amyloid beta deposits [25]. Thus, we first investigated whether platelet depletion changed amyloid plaque pathology in APP-PS1 mice. To measure amyloid plaque burden, we used a semi-automatic method for quantifying thioflavin S-stained plaques in the hippocampus and cortex (Figure 2A) [43, 44]. Platelet-depleted mice showed similar hippocampal and cortical amyloid plaque loads (% of thioflavin S staining), average plaque size and plaque density (number of plaques/area) compared with control IgG treated mice (Supplementary Figure 2A–2C). As APP-PS1 female mice develop a stronger amyloid pathology than males [45], we investigated potential sex-specific effects of platelet depletion on amyloid plaque deposition (Supplementary Figure 2D–2I and Figure 2B–2D). Upon platelet depletion, females showed a significant shift in amyloid plaque size distribution in the hippocampus, presenting fewer small (30–100 μm^2^) and more medium (100–500 μm^2^) size plaques than control females (Figure 2B, 2C). Platelet-depleted females also presented a lower percentage of small plaques (Figure 2B, 2C) and a higher average plaque area in the somatosensory (SS) and somatomotor (MO) cortex (Supplementary Figure 2E) compared with control females. No changes in plaque pathology were observed in males (Supplementary Figure 2G–2I, Figure 2B, 2D).

### Increased neuritic dystrophy around amyloid plaques in platelet-depleted APP-PS1 females

**Figure 3:**
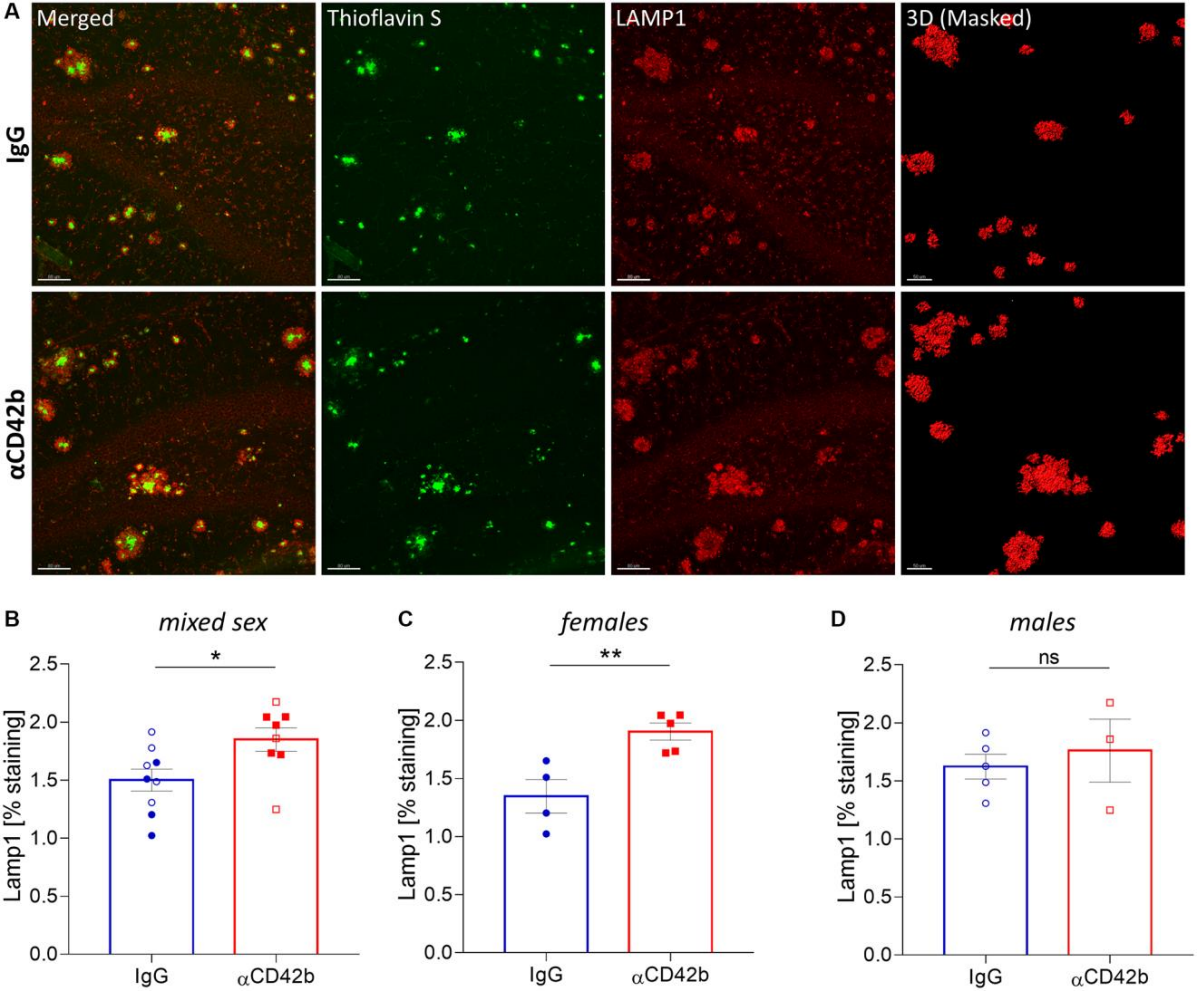
**Platelet depletion increases neuritic dystrophy in the hippocampus of APP-PS1 females.** (**A**) Brain tissue stained with thioflavin S (green, amyloid plaques) and LAMP1 (red, dystrophic neurites) was used for 3D modelling to quantify the volume of LAMP1+ clusters surrounding thioflavin S+ amyloid plaques. (**B**–**D**) Platelet depletion significantly increased plaque-associated dystrophic neurites in females but not in males. Data are shown as mean ± SEM. Statistical analysis was performed by unpaired Student’s *t* test (*n* = 8–9/ treatment; “full forms” represent females and “empty forms” males). ^**^p < 0.01; ^*^p < 0.05. Scale bar: 80 μm and 50 μm (3D mask).

Neuritic pathology correlates with amyloid plaque size, such that larger plaques are accompanied by higher amounts of dystrophic neurites [9, 46]. Thus, we decided to analyze whether platelet depletion also affected dystrophic neurites. For that, we immunolabelled brain tissue for lysosome-associated membrane protein 1 (LAMP1) - a lysosomal protein highly enriched in dystrophic neurites and a common marker of neuronal dystrophy [9, 47] - and quantified the volume of LAMP1+ clusters around thioflavin S+ plaques in the hippocampus (Figure 3A). Platelet-depleted mice showed a significant increase in plaque-associated neuritic dystrophy compared with IgG treated controls (Figure 3B). However, when we analyzed sex-specific effects, only females showed significantly increased levels of neuritic dystrophy with platelet depletion (Figure 3C, 3D).

### Microglia phagocytosis is altered in platelet-depleted APP-PS1 females

**Figure 4:**
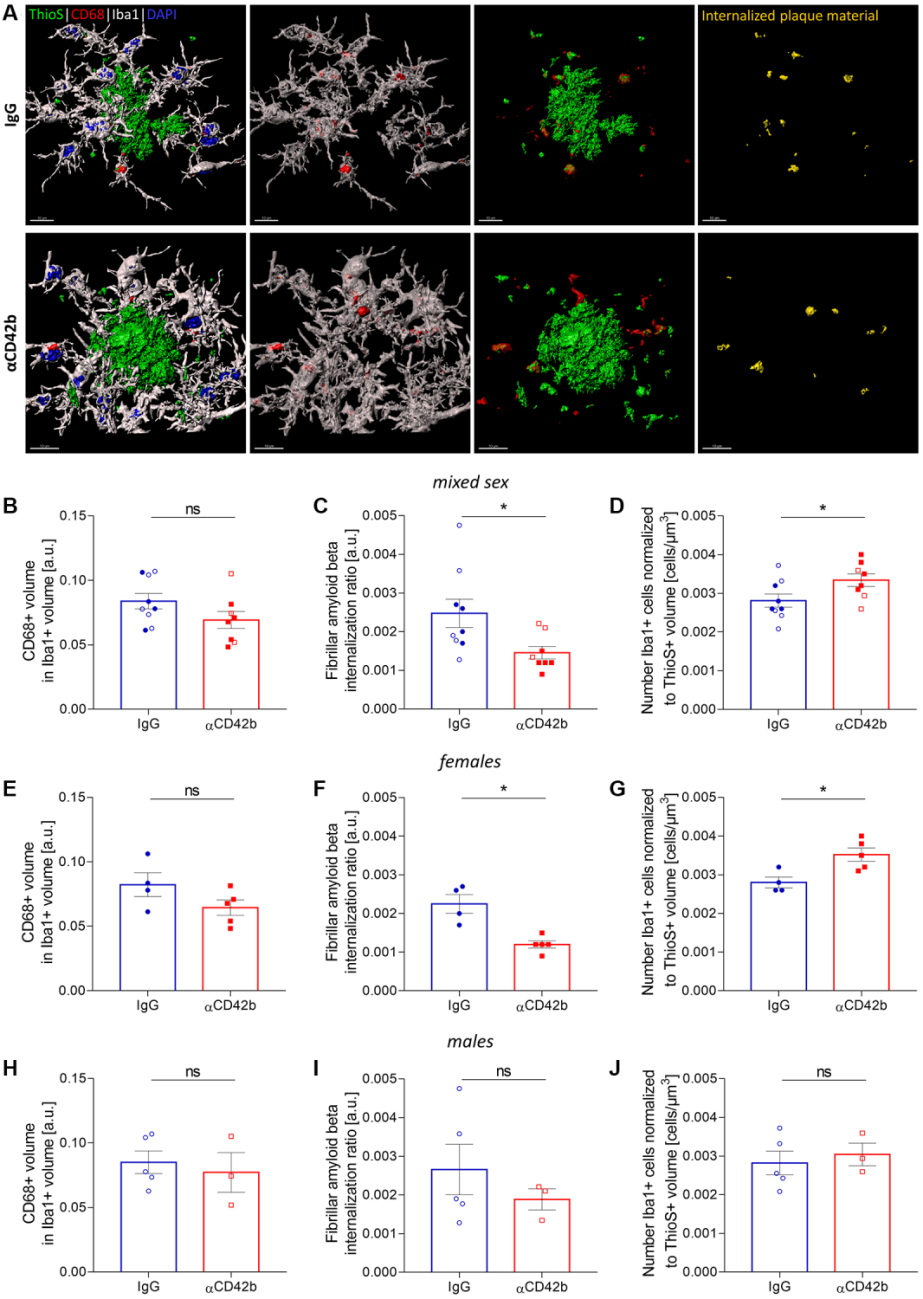
**Platelet depletion impairs amyloid beta phagocytosis by microglia.** (**A**) Brain tissue stained for amyloid plaques (green, thioflavin S), microglia (white, Iba1) and microglial phagolysosomes (red, CD68) was used for 3D *in silico* analysis of microglial phagocytosis in the hippocampus. (**B**–**D**) Platelet-depleted mice showed similar CD68+ phagolysosome volume, decreased amounts of internalized fibrillar amyloid beta and a higher number of plaque-associated microglia compared with IgG treated controls. (**E**–**G**) The same effects were observed in platelet-depleted females. (**H**–**J**) Males showed no significant differences between experimental groups. Data are shown as mean ± SEM. Statistical analysis was performed by unpaired Student’s *t* test (*n* = 8–9/treatment; “full forms” represent females and “empty forms” males). ^*^p < 0.05. Scale bar: 10 μm. Abbreviation: ThioS: thioflavin S.

In AD, microglial cells cluster around amyloid plaques, where they actively phagocytose fibrillar amyloid beta and modulate plaque compaction, thereby preventing neuronal damage [11, 12, 48, 49]. Given the effects of platelet depletion on amyloid plaque pathology and neuritic dystrophy, we decided to investigate whether microglial function was altered. We used confocal imaging and 3D surface rendering to measure the microglial cargo of thioflavin S-labelled plaque material [48, 50, 51]. For that, we volumetrically quantified the signal of thioflavin S+ colocalized within CD68-immunolabeled phagolysosomes of plaque-associated Iba1+ microglia in the hippocampus (Figure 4A–4J). Microglia from platelet-depleted mice showed similar CD68+ phagolysosome volume (Figure 4B) compared with control mice but lower amounts of internalized amyloid plaque content (Figure 4C) and increased numbers of plaque-associated Iba1+ microglia (Figure 4D). When analyzing sex-specific effects, we observed the same effects in platelet-depleted females (Figure 4E–4G), whereas males showed no statistically significant differences between experimental groups (Figure 4H–4J).

### Platelet depletion increases astrocytic coverage of fibrillary amyloid plaques

**Figure 5:**
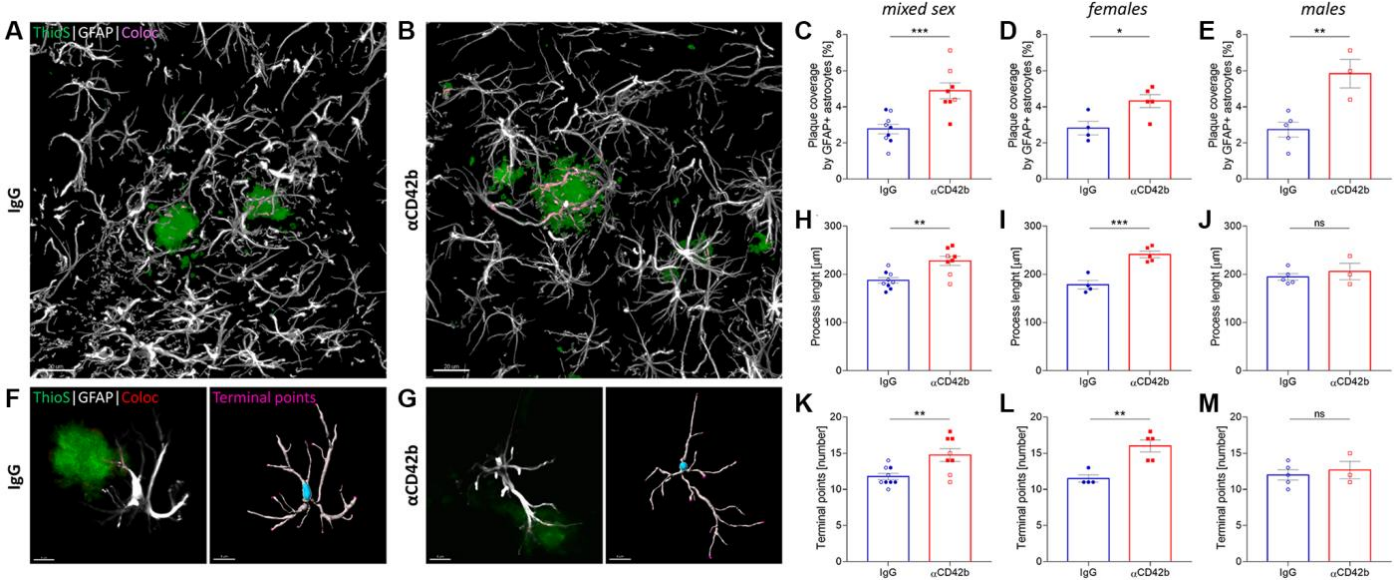
**Platelet depletion increases astrocytic coverage of fibrillary amyloid plaques.** (**A**, **B**) Brain tissue stained for amyloid plaques (green, thioflavin S) and astrocytes (white, GFAP) was used to analyze astrocyte-plaque interactions in the hippocampus. (**C**) Platelet-depleted mice showed increased overlap between astrocytic processes and amyloid plaques. (**D**, **E**) This effect was observed independent of sex. (**F**, **G**) Morphological analysis of plaque-associated astrocytes revealed increased (**H**–**J**) process length and (**K**–**M**) branching in platelet-depleted females but not in males. Data are shown as mean ± SEM. Statistical analysis was performed by unpaired Student’s *t* test (*n* = 8–9/group; “full forms” represent females and “empty forms” males). ^***^p < 0.001; ^**^p < 0.01; ^*^p < 0.05. Scale bar: 10 μm. Abbreviation: ThioS: thioflavin S.

Alongside microglia, astrocytes also play a key role in amyloid plaque dynamics. Hypertrophic astrocytes enwrap and invade amyloid deposits with their processes [52], limiting plaque growth and neuritic dystrophy [53]. To analyze the impact of platelet depletion in the astrocyte-plaque interaction, we used colocalization studies and 3D surface rendering to assess the overlap between GFAP immunolabeled astrocytes and thioflavin S+ plaques (Figure 5A–5E) and the morphology of plaque-associated astrocytes (Figure 5F–5M). Platelet-depleted mice showed increased coverage of hippocampal thioflavin S+ plaques by astrocytic processes (Figure 5C–5E) and a higher total astrocyte process length and branching (i.e., number of branch endings) compared with control mice (Figure 5H, 5K). However, analysis of sex-specific effects revealed significant differences in astrocyte morphology only in females (Figure 5I, 5J, 5L, 5M).

## DISCUSSION

Platelets are the primary source of amyloid beta in the blood [22] and seem to fuel cerebrovascular [24, 26, 28, 29] and cerebral amyloid beta deposition in animal models [25], leading us (and others [30]) to hypothesize that reducing platelet numbers would alleviate AD neuropathology. However, the present study shows that short-term platelet depletion augments amyloid plaque growth, increases neuritic dystrophy, impairs microglial phagocytosis and changes astrocytic morphology in the hippocampus of one-year-old APP-PS1 female mice. Platelet-depleted mice, irrespective of sex, also showed higher coverage of amyloid plaques by astrocytic processes, indicating alterations in astrocyte-amyloid plaque interaction. Thus, platelets might have protective functions at advanced stages of amyloid plaque pathology by either directly influencing amyloid plaque deposition or modulating glial cell function.

Amyloid plaques are dynamic structures, and changes in plaque size occur within days, even in advanced disease stages [54–57]. After 5-days of thrombocytopenia, the size distribution of amyloid plaques in the hippocampus of APP-PS1 female mice shifted towards bigger plaques (i.e., the percentage of small plaques decreased, and the percentage of medium plaques increased), but amyloid plaque load and density remained stable. Platelet-depleted APP-PS1 females also showed a lower percentage of small plaques and a bigger average plaque area in the somatomotor and somatosensory cortex. Based on our observations, we hypothesize that platelet depletion shifted amyloid plaque size by mainly promoting the growth of small-size plaques, as these have the fastest growth rates [54]. The growth of small-size plaques combined with the rare plaque formation characteristic of advanced disease stages [55, 58, 59] could explain the decrease in the frequency of smaller plaques and the presence of bigger plaques without changes in plaque density. Small plaque growth seems to induce higher levels of neuritic damage [47], which could explain the increase in plaque-associated neuritic dystrophy in the hippocampus of platelet-depleted APP-PS1 female mice.

Our study suggests that platelets might contribute to limiting amyloid plaque size and neuronal damage, having a beneficial role in AD. However, previous animal studies suggest that platelets support amyloid plaque formation and neuroinflammation [24–26]. Differences in the disease stage (i.e., early versus advanced stage) and the animal models investigated might account for these contradictory observations. For example, Wu et al. [25] reported that WT mice injected with APP-PS1 mouse platelets develop amyloid plaque pathology [25], suggesting that platelet-derived amyloid beta contributes to plaque seeding – a concept further supported by *in vitro* studies showing that platelets release amyloid beta and promote its fibrilization [24, 28, 60, 61]. Like Wu et al. [25], we worked with APP-PS1 mice, but instead of investigating a pre-amyloid plaque stage, we performed our experiments in mice with a severe amyloid plaque load. Donner et al. [24] and Khalaf et al. [62] showed that clopidogrel, a platelet inhibitor, reduces cerebrovascular amyloid beta deposition in APP23 mice [24] and hippocampal amyloid beta deposition in aluminum-induced AD rats [62]. However, the kinetics of amyloid beta deposition differs across models and for example, APP-PS1 mice have an accelerated amyloid pathology compared to age-matched APP23 mice [63]. Future experiments assessing the effects of platelet depletion at different disease stages will help to better understand how platelets influence amyloid beta pathology.

Given that platelet depletion changes amyloid plaque size, important questions arise: How do platelets modulate the growth kinetics of amyloid plaques? What are the implications of plaque growth in platelet-depleted APP-PS1 mice? Previous mouse studies directly linked platelet-derived factors such as amyloid beta [25, 26] and clusterin [24] with amyloid plaque formation. However, the accelerated plaque growth in platelet-depleted APP-PS1 female mice suggests that platelets influence amyloid plaque pathology through alternative mechanisms.

To better understand the effects of platelet depletion on AD pathology, we investigated plaque-associated microgliosis and astrogliosis. Reactive microglia and astrocytes cluster around amyloid plaques [52, 64, 65] and are critical mediators of amyloid beta pathogenesis [11, 12, 48, 49, 52, 53]. Microglia phagocytose amyloid beta and are involved in plaque compaction, limiting plaque size and neurotoxicity [11, 48]. Astrocytes enwrap amyloid plaques with their processes [52], a mechanism also involved in restricting plaque growth [53]. Thus, dysregulation of glial function during platelet depletion could contribute to changes in amyloid plaque pathology.

After platelet depletion, APP-PS1 females had more plaque-associated microglia, but these cells showed lower fibrillar amyloid cargo in their phagolysosomes, suggesting that microglial phagocytosis was less efficient. Impaired amyloid plaque clearance by microglia could justify the growth of amyloid plaques after platelet depletion. However, Huang et al. [48] recently demonstrated that microglial phagocytosis is crucial for dense-core plaque formation and suggested that microglia work as “factories” of amyloid beta fibrillization: microglia uptake amyloid beta and convert it into insoluble fibrils, which upon release, fuel amyloid plaque growth [48]. Thus, an alternative hypothesis is that platelet depletion enhances the secretion of amyloid fibrils by microglia, resulting in lower phagolysosome cargo and providing amyloid fibrillar material for plaque growth.

In APP-PS1 mice, platelet depletion increased the overlap between astrocytic processes and amyloid plaques. Platelet-depleted females also showed morphological alterations suggestive of increased astrocyte reactivity. In females, changes in astrocyte-amyloid plaque interaction could be seen as a compensatory response: plaque growth and dysregulated microglia function might trigger astrocytes to enwrap amyloid plaques more to restrict plaque growth. However, as platelet-depleted males, which showed no changes in plaque pathology or astrocytic morphology, also had an enhanced astrocyte-plaque interaction, the effects of platelet depletion on astrogliosis might be independent of changes in plaque pathology.

Evidence from different murine models suggests that platelets and platelet-derived factors directly interact with glial cells, modulating neuroinflammation [41, 66–70]. Injections of AD transgenic mouse platelets into wild-type mice induce microglial activation [25, 26]; and in APP-PS1 mice, intra-parenchymal platelets closely associate with astrocytes [71]. The molecular mechanisms underlying the effects of platelet depletion on glial function and its biological implications for AD pathology remain elusive and need to be assessed in future experiments.

An alternative hypothesis, not investigated by us, is that platelet depletion affects amyloid plaque pathology and gliosis in APP-PS1 mice due to changes in vascular permeability. Platelets are essential for vascular integrity, and thrombocytopenia leads to changes in vascular permeability [72–74]. Leakage of neurotoxic and neuroinflammatory blood components into the brain and/or a faulty clearance of waste products (such as amyloid beta peptides) from the brain could also lead to the worsening of AD neuropathology in APP-PS1 mice.

In our study, platelet depletion seems to affect AD pathology differentially in female and male APP-PS1 mice. APP-PS1 mice show sex-related differences in amyloid pathology [45, 75–78] and microglia [76], which could explain the sex differences observed after platelet depletion. An alternative hypothesis is that platelet depletion leads to sex-specific changes in the systemic milieu that favor amyloid plaque formation in females. Upon activation, platelets release multiple bioactive mediators [79, 80]. Female platelets are more reactive than male platelets [81, 82] and the composition of platelet-rich plasma (plasma enriched in platelets and depleted of other blood cells by centrifugation) seems to differ between women and men [83]. Although females and males showed similar depletion efficiencies and the anti-platelet antibody injected does not activate platelets [39], sex differences in platelet cargo could lead to differences in blood circulating factors. However, it is unknown whether sexual dimorphism affects APP-PS1 mouse platelets (or AD platelets in general) or whether platelet depletion affects plasma composition or the brain environment.

Platelets contain several mediators with the potential to modulate brain functioning [25, 41, 68–70, 84, 85]; and seem to be involved in neuroinflammation [41, 66–70], neuronal electric activity [84], adult neurogenesis [85] and synaptic plasticity [86]. In APP-PS1 females, acute thrombocytopenia aggravates AD neuropathology, suggesting that platelets might have a protective function in AD. However, the underlying molecular mechanisms by which platelets modulate amyloid plaque deposition remain elusive and need to be investigated in future experiments.

## METHODS

### Animals

For this study, we used female and male APP Swedish PS1dE9 (APP-PS1) mice expressing a chimeric mouse/human mutant amyloid precursor protein (Mo/HuAPP695swe) and a mutant presenilin 1 (PS1-dE9) under the control of the mouse prion protein promoter (Mutant Mouse Resource and Research Center (MMRRC) strain #034832-JAX, The Jackson Laboratory) [45, 87]. Prion protein promoter directs transgene expression mainly to central nervous system neurons [87], but also to the megakaryocyte lineage [21, 88]. Mice were housed in groups, in individually ventilated cages (IVC), under standard conditions at the Paracelsus Medical University with a constant 12-hour light/dark cycle, at 22°C, RT and with *ad libitum* access to standard rodent chow (sniff^®^ rodent maintenance chow 10mm pellets (#V1534)) and water. Animal breeding, handling, genotyping, and experiments were approved by local authorities (BMWFW-66.019/0011-WF/V/3b/2016 and 2020-0.078.469). Animals were bred under specific pathogen free (SPF) conditions and transferred in IVC to non-SPF conditions for platelet depletion experiments.

### Platelet depletion experiment

To induce thrombocytopenia, 12 to 13-months old APP-PS1 mice (*n* = 8–9/group) were treated with an antibody preparation of rat purified monoclonal antibodies directed against mouse glycoprotein (GP) Ibα (CD42b) (#R300, Emfret Analytics) or non-immune rat immunoglobulins (IgG) (#C301, Emfret Analytics). Two intraperitoneal injections (50 μg of antibody in 100 μL) were administered three days apart (i.e., experimental days 1 and 4). The appropriate dose of αCD42b to achieve severe thrombocytopenia was established after initial titrations (data not shown). On experimental day 5, animals were sacrificed for tissue and blood sampling.

To monitor platelet depletion efficacy, blood was collected from the lateral saphenous vein five days before (baseline platelet count) and 48 hours after the first injection (experimental day 3). For that, animals were restrained and punctured with a BD Microlance 26G × 1/2” syringe needle. A blood sample of about 20 to 30 μL was collected using a Minivette^®^ POCT K3 EDTA (#17.2113.050, SARSTEDT AG & Co. KG) and added to ethylenediaminetetraacetic acid (EDTA, 0.1 M, Promega) to further prevent blood coagulation. Additionally, platelet counts were monitored on the last day of the experiment using blood collected by cardiac puncture. Blood samples were analyzed by flow cytometry and using an automated hematology analyzer (Sysmex pocH-100iV Diff; Sysmex Europe GmbH) to determine platelet counts and other hematologic parameters.

### Flow cytometry for platelet count monitoring

Platelet counts were determined by flow cytometry as previously described by others [89]. Briefly, 20 μL of EDTA anticoagulated whole blood were diluted 1:20 in PBS-BSA buffer [sodium phosphate buffer (PBS) Dulbecco (Merck) containing 0.1% bovine serum albumin (BSA, Sigma)]. A 50 μL aliquot of diluted blood was stained with allophycocyanin (APC)-labelled anti-mouse CD41 antibody (1:100, #133913, Biolegend) for 20 min in the dark. The staining reaction was stopped by sample dilution with PBS-BSA buffer (final sample dilution 1:1000). To ensure homogeneity, samples were mixed by inversion and immediately analyzed on the BD Accuri TM C6 Plus flow cytometer (BD Biosciences). Samples were analyzed at medium speed (Flow rate: 35 μl/min) using an FSC-H threshold of 25000 to exclude debris, without losing platelet events. The platelet population was defined based on CD41 staining and forward scattering pattern on a logarithmic scale. A minimum of 25 000 CD41+ events were collected per sample.

### Tissue sampling and processing

Mice were anaesthetized with a solution of ketamine (20.5 mg/mL, Ketamidor, Richter Pharma), xylazine (5.36 mg/mL, Chanazine, Chanelle) and acepromazine (0.27 mg/mL, Vanastress 10 mg/mL, Vana GmbH) in 0.9% sodium chloride. Following thoracotomy, blood was collected by cardiac puncture using EDTA (0.1 M) coated syringes and BD Microlance 23G × 1” needles. To further prevent blood coagulation, EDTA (0.1 M) was added to all samples at a 1:10 ratio. Brains were extracted without perfusion and post-fixed by immersion in 4% paraformaldehyde (in 0.1 M sodium phosphate solution; pH = 7.4) for 4 hours at RT. Afterwards, brains were cryopreserved in PBS containing 0.05% sodium azide and transferred into 30% sucrose (in 0.1 M sodium phosphate solution; pH = 7) before sectioning. Brains were cut on dry ice into 40 μm thick sagittal sections using a sliding microtome (Leica) and stored at −20°C.

### Fluorescence immunohistochemistry

Fluorescence microscopy was performed on free-floating slices as previously described [34, 90]. Briefly, brain sections were washed in PBS and incubated in preboiled 1× citrate buffer (pH = 6.0, Sigma) for 12 min for heat induced antigen retrieval. After washing in PBS, tissue was blocked and permeabilized with fish skin gelatin buffer [PBS containing 1% BSA (Sigma); 0.2% fish skin gelatin (Sigma); 0.1% triton-X (Sigma)] for 1 h at RT. For staining, tissue was incubated with primary antibodies overnight at RT with constant agitation. The following primary antibodies were used: biotin rat monoclonal anti-CD41 [clone MWReg30] (1:300, ab95727, Abcam); rabbit polyclonal anti-LAMP1 (1:500, ab24170, Abcam); goat polyclonal anti-Iba1 (1:500, ab5076, Abcam); rabbit polyclonal anti-CD68 (1:300, ab125212, Abcam); guinea pig polyclonal anti-GFAP (1:500, GP52, Progen). After extensive washing in PBS, tissue was incubated for 3 hours with secondary antibodies (all diluted 1:1000). The following secondary antibodies were used: streptavidin Alexa 568 (21832, Fisher Scientific); anti-rabbit Alexa 647 (A31573, Invitrogen); anti-goat Alexa 647 (705-605-147, Jackson), anti-rabbit Alexa 568 (A10042, Invitrogen); anti-guinea pig Alexa 647 (706-605-148, Jackson). For amyloid beta plaque staining, thioflavin S (1 mg/mL, 1:625, Sigma) was added to the secondary antibody solution. Nucleus counterstaining was performed with 4’-6’-diamidino-2-phenylindole dihydrochloride hydrate (DAPI; 1 mg/mL, 1:2000; Sigma).

### Microscopy and image processing

Mouse brain tissue was imaged using a confocal laser scanning microscope LSM 710 (Zeiss) or VS120 Virtual-Slide-Microscope (Olympus) as further specified. For image analysis, ImageJ/Fiji (version 2.1.0/1.53 h) and Imaris (Bitplane, version 9.3.1, and 9.9.0) were used.

### Analysis of immunohistochemical data

To quantify platelet numbers in the brain, four confocal z-stack images of different hippocampi (objective magnification 20×, zoom 0.6) and cortices (objective magnification 40×, zoom 0.6) were acquired per animal, *n* = 8–9/group). Platelets, visualized by CD41 immunostaining, were counted using ImageJ’s plugin Cell Counter.

To analyze amyloid plaque pathology, four entire sagittal brain sections (with a visible hippocampus and cortex) were stained with thioflavin S and imaged with a VS120 microscope (objective magnification 20×, *n* = 8–9/group). Images were segmented using ImageJ’s plugin ImageSURF [43, 44] and analyzed using the “Analyze Particles” function in ImageJ/Fiji. The following measures were obtained: percentage area of thioflavin S staining, number of plaques per mm^2^, individual and average plaque area. To analyze plaque size distribution, the size of individual plaques was used to determine the percentage of small (30–100 μm^2^), medium (100–500 μm^2^), and large (>500 μm^2^) plaques. A minimum size threshold was set at 30 μm^2^ to exclude possible staining artefacts. Plaque pathology was analyzed in the entire hippocampal and cortical areas and, to assess regional differences in cortical amyloid beta deposition, in two regions of interest in the cortex (i.e., somatosensory and somatomotor cortex).

To quantify plaque-associated neuritic dystrophy, four confocal z-stack images of different hippocampi were acquired per animal (objective magnification 20×, zoom 0.6, *n* = 8–9/ group) and used for 3D modelling. Imaris software was used to mask the clusters of LAMP1 staining around thioflavin S amyloid beta plaques and to measure the volume of these structures.

To analyze microglia phagocytosis, five to seven z-stack images of thioflavin S labelled amyloid plaques located in different hippocampi were acquired per animal (objective magnification 63×, zoom 0.8, *n* = 8–9/group) and used for 3D modelling with Imaris software as previously described [50, 51]. To calculate the ratio of amyloid beta (Abeta) internalization by microglia, the volume of thioflavin S staining colocalized within CD68+ phagolysosomes was normalized to the number of plaque-associated Iba1+ microglia cells and plaque volume. Plaque-associated microglia were manually classified as cells with processes closely associated with amyloid plaques. Only microglia cells with a cell nucleus and visible soma were included in the analysis, and microglia processes without a visible connection to a soma were excluded.

To investigate the association between astrocytes and amyloid plaques and astrocyte morphology, four confocal z-stacks images of different hippocampi were acquired per animal (objective 63×, zoom 0.6, *n* = 8–9/group). Astrocyte coverage of amyloid plaques was analyzed using Imaris colocalization function. Morphological characterization of plaque-associated astrocytes (i.e., cells with processes closely associated with amyloid plaques) was performed using Imaris filament tracer function, using the “Autopath (no loops)” algorithm. Cell models (10 cells/animal) were created automatically but false positive branches and connections were eliminated manually. The corrected models were used to calculate the total filament length and branching terminal points.

### Statistics

Statistical analysis was performed using Grap Prism (Version 9.3.0). Data were tested for normality using the Shapiro-Wilk normality test. To compare values between two groups, unpaired Student’s *t*-test was used for normally distributed data and Mann-Whitney test for non-normally distributed data. For multiple comparisons between groups, one-way or two-way ANOVA with Tukey’s or Holm-Šídák’s multiple comparison tests were used as specified in the figure legends. Data are depicted as mean ± standard error of the mean (SEM) or mean ± standard deviation (SD) as specified in the figure and table legends.

## Supplementary Materials

Supplementary Figures

Supplementary Tables
