# Supplementary material for: Immune-mediated platelet depletion augments Alzheimer’s disease neuropathological hallmarks in APP-PS1 mice
Source: Aging (Albany NY). 2023 Feb 1;15(3):630–49. doi: 10.18632/aging.204502 (PMC9970308; doi:10.18632/aging.204502)
Supplement: Supplementary Figures [file aging-15-204502-s001.pdf]

SUPPLEMENTARY FIGURES

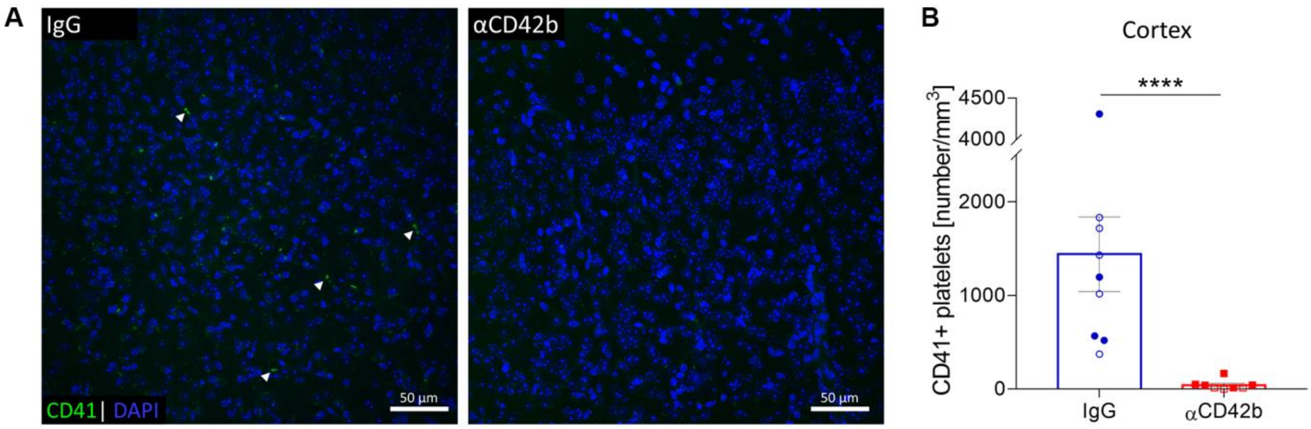

Supplementary Figure 1. **Analysis of platelet density in brain cortical tissue upon platelet depletion.** (**A**) Brain tissue immunolabelled for CD41 (green) was used to quantify platelets in the cortex. DAPI (blue) was used as nucleus staining. (**B**) Platelets were significantly reduced in the cortex of platelet-depleted mice. Data are shown as mean ± SEM. Statistical analysis was performed by unpaired Student's *t* test (*n* = 8–9/treatment; "full forms" represent females and "empty forms" males). $^{****}p < 0.0001$. Scale bar: 50 µm.

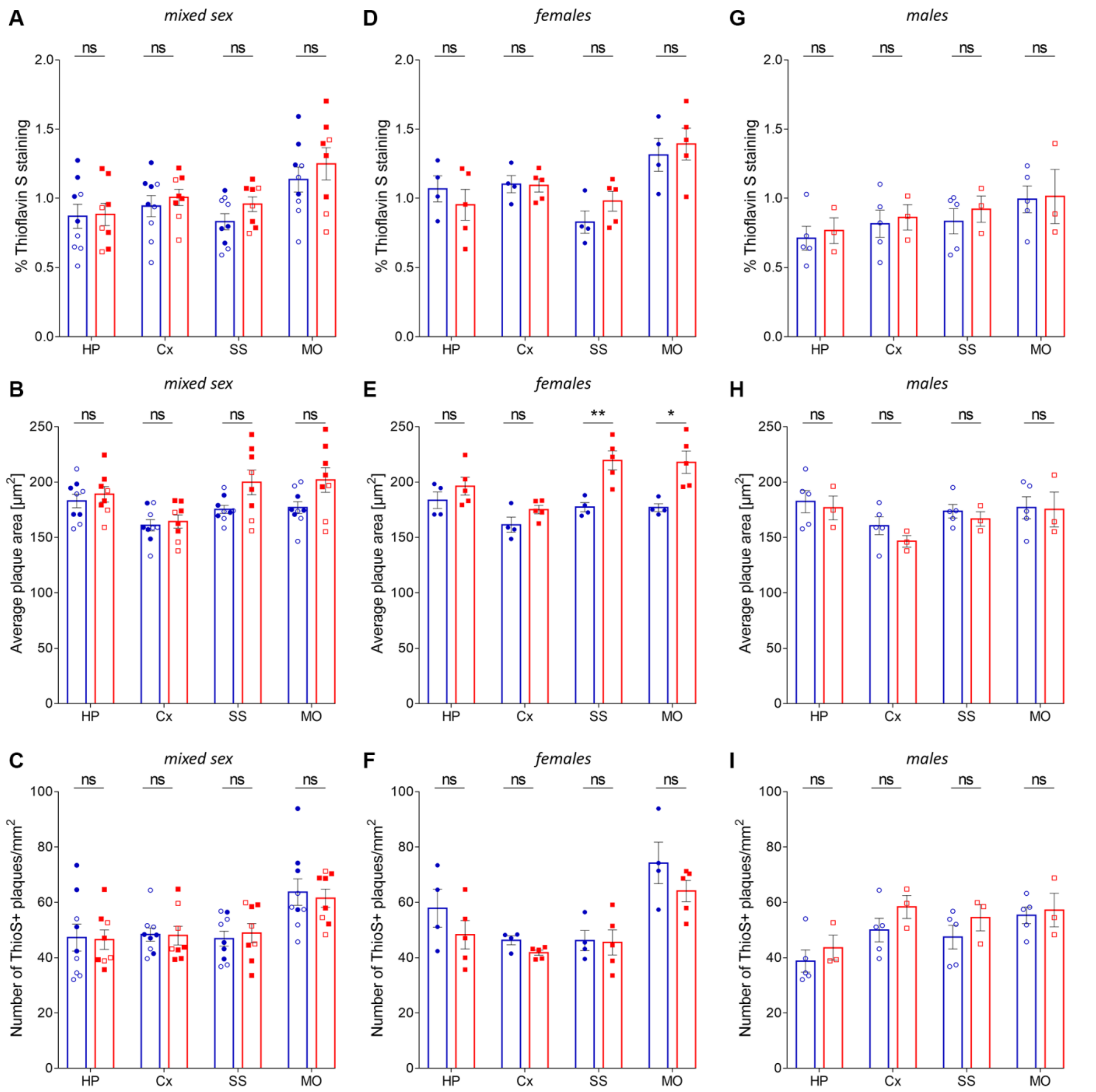

Supplementary Figure 2. Analysis of amyloid plaque pathology in APP-PS1 mice upon platelet depletion. Amyloid plaque load was assessed in the hippocampus (HP), whole cortex (Cx), somatosensory (SS) and somatomotor (MO) cortex of thioflavin S (ThioS) labelled brain sections. (**A**–**C**) No changes were observed in amyloid load (% thioflavin S staining), average plaque size or plaque density after platelet depletion. (**D**–**I**) Sex-specific effects analysis revealed a significant increase in average plaque area in SS and MO cortex in platelet-depleted females. No significant differences were observed in males. Data are shown as mean ± SEM. Statistical analysis was performed by ordinary two-way ANOVA with Šídák's multiple comparisons test (n = 8–9/treatment; "full forms" represent females and "empty forms" males). $^{**}p < 0.01$; $^{*}p < 0.05$. "full forms" represent females and "empty forms" males.
