# Supplementary material for: Immune-mediated platelet depletion augments Alzheimer’s disease neuropathological hallmarks in APP-PS1 mice
Source: Aging (Albany NY). 2023 Feb 1;15(3):630–49. doi: 10.18632/aging.204502 (PMC9970308; doi:10.18632/aging.204502)
Supplement: Supplementary Tables [file aging-15-204502-s002.pdf]

SUPPLEMENTARY TABLES

Supplementary Table 1. Hematological data of platelet-depleted and control APP-PS1 mice on day 3.

	Mixed Sex			Females			Males		
	**IgG**	**αCD42b**	***p* value**	**IgG**	**αCD42b**	***p* value (adj.)**	**IgG**	**αCD42b**	***p* value (adj.)**
PLT ($x10^3$/μl)	**1545.33 ± 362.51**	**21.49 ± 16.64**	**<0.0001**	**1352.00 ± 494.43**	**18.81 ± 14.92**	**<0.0001**	**1700.00 ± 112.70**	**25.97 ± 21.82**	**<0.0001**
WBC ($x10^3$/μl)	16.50 ± 5.58	15.59 ± 4.30	0.7145	11.98 ± 3.86	12.85 ±2.52	0.9758	20.12 ± 3.78	20.16 ± 1.40	>0.9999
RBC ($X10^6$/μl)	12.40 ± 1.28	12.23 ± 0.67	0.7267	13.25 ± 0.88	12.44 ± 0.46	0.5563	11.73 ± 1.19	11.87 ± 0.92	0.9967
HCT (%)	32.77 ± 2.91	34.11 ± 1.38	0.2514	33.75 ± 3.16	34.08 ± 1.06	0.9967	31.98 ± 2.77	34.17 ± 2.10	0.6046
HGB (g/dl)	17.77 ± 1.58	17.27 ± 0.71	0.4244	18.60 ± 1.45	17.68 ± 0.47	0.6249	17.11 ± 1.48	16.6 ± 0.48	0.921
MCV (fl)	42.21 ± 1.59	41.9 ± 1.07	0.6472	41.25 ± 0.59	41.68 ± 0.35	0.9563	42.98 ± 1.76	42.27 ± 1.86	0.8663
MCH (pg)	14.36 ± 0.55	14.05 ± 0.38	0.209	14.05 ± 0.31	14.06 ± 0.18	>0.9999	14.60 ± 0.60	14.03 ± 0.67	0.3713
MCHC (g/dl)	**54.36 ± 3.70**	**50.6 ± 1.98**	**0.0217**	55.18 ± 1.92	51.80 ± 0.70	0.3582	53.70 ±4.84	48.59 ± 1.78	0.1311
Sample size	9	8		4	5		5	3	

Blood parameters were assessed in whole blood collected from the lateral saphenous vein 48 hours after the first anti-CD42b antibody injection and analyzed using a hematology analyzer. Results are presented as mean ± SD (*n* = 8–9 mice/group). Statistical analysis was performed by unpaired Student's *t*-test (mixed sex) or one-way ANOVA with Tukey's multiple comparisons test (sex-specific effects). IgG: control group treated with non-immune immunoglobulins. αCD42: platelet-depleted group treated anti-CD42b antibodies. Abbreviations: WBC: white blood cells; RBC: red blood cells; HCT: hematocrit; HGB: hemoglobin, MCV: mean corpuscular volume; MCH: mean corpuscular hemoglobin; MCHC: mean corpuscular hemoglobin concentration.

Supplementary Table 2. Hematological and weight data of platelet-depleted and control APP-PS1 mice on day 5.

	Mixed Sex			Females			Males		
	**IgG**	**αCD42b**	***p* value**	**IgG**	**αCD42b**	***p* value (adj.)**	**IgG**	**αCD42b**	***p* value (adj.)**
PLT ($x10^3$/μl)	**1198.11 ± 311.02**	**7.18 ± 7.79**	**<0.0001**	**1194.00 ± 144.97**	**7.82 ± 9.03**	**<0.0001**	**1261.83 ± 397.33**	**3.5 ± 3.6**	**<0.0001**
WBC ($x10^3$/μl)	1.73 ± 0.77	1.88 ± 0.67	0.7548	1.53 ± 0.31	2.16 ± 0.69	0.54	1.97 ± 0.96	1.43 ± 0.29	0.7039
RBC ($X10^6$/μl)	**8.56 ± 1.12**	**6.67 ± 2.23**	**0.0409**	8.97 ± 1.43	7.37 ± 1.91	0.5196	8.23 ± 8.40	5.51 ± 2.65	0.1769
HCT (%)	29.64 ± 5.97	24.76 ± 7.76	0.1639	32.73 ± 5.79	27.32 ± 7.53	0.6269	27.18 ± 5.40	20.90 ± 7.90	0.5788
HGB (g/dl)	**12.04 ± 1.61**	**9.41 ± 3.12**	**0.0417**	12.35 ± 2.24	10.46 ± 2.65	0.6461	11.80 ± 1.14	7.67 ± 3.55	0.1301
MCV (fl)	**42.44 ± 1.41**	**44.60 ± 2.36**	**0.0351**	42.18 ± 0.61	44.78 ± 2.51	0.3909	42.66 ± 1.89	44.30 ± 2.59	0.6434
MCH (pg)	14.08 ± 0.49	14.16 ± 0.46	0.7199	13.75 ± 0.34	14.22 ± 0.46	0.4288	14.34 ± 0.45	14.07 ± 0.55	0.8358
MCHC (g/dl)	41.95 ± 9.72	37.93 ± 3.79	0.6548	37.73 ± 1.04	39.00 ± 3.95	0.9527	45.34 ± 12.48	36.13 ± 3.39	0.5102
Weight variation (% of initial weight)	0.19 ± 1.93	−0.99 ± 2.24	0.2621	−0.83 ± 1.87	−0.002 ± 2.17	0.6502	0.98 ± 1.73	−2.64 ± 1.26	0.1056
Sample size	9	8		4	5		5	3	

Blood parameters were assessed in whole blood collected by cardiac puncture on the final day of the experiment using a hematology analyzer. Weight variation represents the percentage of body weight variation during the experiment. Results are presented as mean ± SD (*n* = 8–9 mice/group). Statistical analysis was performed by unpaired Student's *t*-test (mixed sex) or one-way ANOVA with Tukey's or Holm-Šídák's multiple comparisons test (sex-specific effects). IgG: control group treated with non-immune immunoglobulins. αCD42: platelet-depleted group treated anti-CD42b antibodies. Abbreviations: WBC: white blood cells; RBC: red blood cells; HCT: hematocrit; HGB: hemoglobin, MCV: mean corpuscular volume; MCH: mean corpuscular hemoglobin; MCHC: mean corpuscular hemoglobin concentration.
